# Characterization of PLA/PBSeT Blends Prepared with Various Hexamethylene Diisocyanate Contents

**DOI:** 10.3390/ma14010197

**Published:** 2021-01-03

**Authors:** Sun Jong Kim, Hyo Won Kwak, Sangwoo Kwon, Hyunho Jang, Su-il Park

**Affiliations:** 1 Department of Packaging, Yonsei University, 1 Yonseidae-gil, Wonju 26493, Korea; 7.7.7.8@yonsei.ac.kr (S.J.K.); ksw0089@naver.com (S.K.); whyhyun@naver.com (H.J.); 2 Department of Agriculture, Forestry and Bioresources, Seoul National University, 1 Gwanak-ro, Gwanak-gu, Seoul 08826, Korea; bk0502@snu.ac.kr

**Keywords:** PBSeT, blend, crosslinking, HDI

## Abstract

Poly (lactic acid) (PLA) is the most widely available commercial bioplastic that is used in various medical and packaging applications and three-dimensional filaments. However, because neat PLA is brittle, it conventionally has been blended with ductile polymers and plasticizers. In this study, PLA was blended with the high-ductility biopolymer poly (butylene-sebacate–*co*–terephthalate) (PBSeT), and hexamethylene diisocyanate (HDI) was applied as a crosslinking compatibilizer to increase the miscibility between the two polymers. PLA (80%) and PBSeT (20%) were combined with various HDI contents in the range 0.1–1.0 parts-per-hundred rubber (phr) to prepare blends, and the resulting physical, thermal, and hydrolysis properties were analyzed. Fourier-transform infrared analysis confirmed that –NH–C=OO^−^ bonds had formed between the HDI and the other polymers and that the chemical bonding had influenced the thermal behavior. All the HDI-treated specimens showed tensile strengths and elongations higher than those of the control. In particular, the 0.3-phr-HDI specimen showed the highest elongation (exceeding 150%) and tensile strength. In addition, all the specimens were hydrolyzed under alkaline conditions, and all the HDI-treated specimens degraded faster than the neat PLA one.

## 1. Introduction

With the increasing urgency to reduce carbon-dioxide emissions, numerous eco-friendly, sustainable, biomass-based materials have been studied recently to manage plastic waste and neutralize CO_2_ emissions in the production of plastics [1,2,3]. Poly (lactic acid) (PLA) is a commercially available 100% biomass-based compostable polymer that has been used for research on medical scaffolds, artificial bone structures, and drug delivery [4,5,6,7] and has been widely used and studied as a packaging material for rigid food containers and as a component of flexible films [8,9,10]. In recent years, PLA has been blended with natural fibers and three-dimensional (3D) filaments to produce materials for commercial application to automotive interior parts [11,12,13,14].

However, despite such applications, PLA also has several disadvantages originating from its physical properties [15,16,17]. For example, despite showing high tensile strength, PLA also shows dramatically low elongation and impact resistance, both of which are major obstacles to the commercial application of neat PLA [15,18,19]. Many studies have explored various methods of decreasing the brittleness and increasing the melt strength of PLA by incorporating plasticizers or chain extenders with neat PLA [19,20,21].

Moreover, other studies have shown that biodegradable elastomeric polymers are good candidates for decreasing PLA brittleness while maintaining biodegradability. For example, although PLA has been blended with high-ductility biodegradable elastomeric polymers such as poly (butylene-adipate–*co*–terephthalate) (PBAT), poly (butylene succinic acid) (PBS), poly (hydroxy alkanoate) (PHA), and polycaprolactone (PCL), success has been limited because PLA and the other biodegradable polymers are immiscible [22,23,24,25].

Various compatibilizers reportedly have improved the miscibility of PLA blended with other binary polymers [26,27,28]. Hexamethylene diisocyanate (HDI) reportedly reacts with other polyesters by forming urethane bonds, thereby increasing the molecular weight and improving the processability and physical properties of PLA [29,30]. HDI has been used to modify the PLA physical properties by increasing the surface energies of PLA and other biodegradable polymers to form a network of chemical bonds within an immiscible binary structure [31,32], and the method can be used to efficiently combine binary or ternary structure.

However, fossil-based biodegradable polymers such as PBAT and PBS have been blended with PLA, thereby clearly decreasing the bio-based content or carbon footprint of the resulting blends [33,34,35]. Kim et al. (2020) recently synthesized the random coblock polyester poly (butylene-sebacate–*co*–terephthalate) (PBSeT) from bio-based sebacic acid, and the resulting amorphous PBSeT showed a high elongation of over 1500% and high ductility [36]. Therefore, blending PBSeT with PLA is expected to decrease the PLA brittleness while preserving the decreased content of the biobased carbon content (BCC).

In this study, blends were developed by blending PLA and PBSeT with various HDI contents to improve ductility of PLA, and the physical properties, thermal behavior, and chemical structure of the blends were investigated. In addition, hydrolysis tests were performed to determine the blend degradability.

## 2. Materials and Methods

### 2.1. Materials

Dimethyl terephthalate (DMT) was purchased from SK Chemicals Co., Ltd. (Seoul, Korea). Sebacic acid (Se) and 1, 4-butanediol (BDO) were obtained from Daejung Chemicals & Metals Co., Ltd. (Siheung, Korea). Titanium tetrabutoxide (TBT) was supplied by Merck KGaA (Darmstadt, Germany) and was used as a catalyst for synthesizing. PLA (4032D) was purchased from NatureWorks LLC (Minnetonka, MN, U.S.A) and it had molecular weight (Mw) of 181,000 (g/mol) and dispersity of 1.89. HDI was obtained from Daejung Chemicals & Metals Co., Ltd. (Siheung, Korea).

### 2.2. Synthesis of PBSeT and Blending

**Figure 1:**
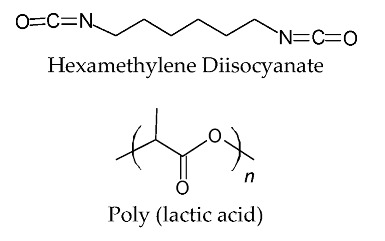

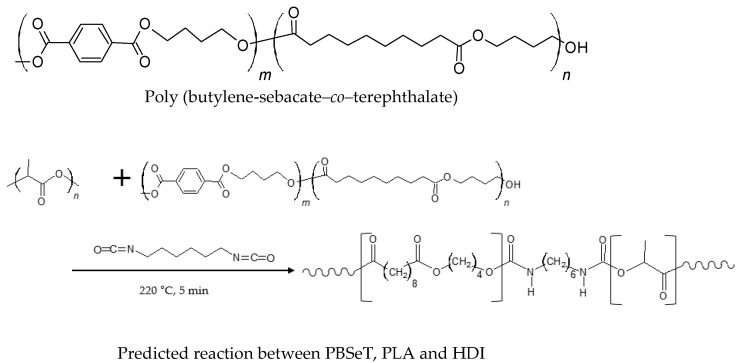
Chemical structures of blend and reactant.

The PBSeT was synthesized using 60 mol% sebacic acid and 40 mol% DMT by esterification and subsequent polycondensation under vacuum in the range 200–240 °C, according to the method described by Kim et al. (2020) [36], and the ratio (mol%) of BDO to dicarboxylic acid was fixed at 1.25:1. The blend was blended using a high-viscosity kneading machine (TEST ONE, Seoul, Korea) at 220 °C for 5 min. The predicted structures of partial blend of the reactants, PBSeT, and blend was shown in Figure 1, and the chemical compositions of the PBSeT/PLA blends prepared with various HDI ratios are listed in Table 1.

### 2.3. Specimen Preparation

Specimens for mechanical testswere injected from an injection-molding machine (GibaeEnT, Gyeonggi, Korea) at 210 °C and 40 MPa for 2 min, according to the ISO 527 standard. Therefore, the temperature of each polymer was set according to the polymer appearance. The specimens for IR analysis and degradation test were hot-pressed (hot-pressing machine, TEST ONE, Seoul, Korea) at 230 °C and 40 MPa for 3 min to a length, width, and thickness of 3, 3, and 0.05 cm, respectively.

### 2.4. Fourier-Transform Infrared Analysis

The Fourier-transform infrared (FTIR) absorption spectra were recorded using an IFS 88-IR spectrometer (Bruker AXS GmbH, Karlsruhe, Germany) in the range 400–4000 cm^−1^ for all the specimens under ambient conditions. The spectral resolution was 2 cm^−1^, and 16 scans were averaged for each specimen.

### 2.5. Mechanical-Property Analysis

The room-temperature (RT) tensile strengths and elongations at break of blends were measured using a universal testing machine (Qmesys, Seoul, Korea) operating at a crosshead speed of 10 mm min^−1^. The test specimens were prepared using a dumbbell-shaped mold manufactured as described in the ISO 527 standard. More than five samples were measured for each polymer, and the mean and standard deviation were calculated.

The 3-point RT bending strengths of the specimens were analyzed using a universal testing machine (Qmesys, Seoul, Korea) operating at a crosshead speed of 10 mm min^−1^, according to the ASTM D790 testing method. More than five samples were measured for each polymer, and the mean and standard deviation were calculated.

### 2.6. Gel-Permeation-Chromatography Analysis

The molecular weights (*M*_n_ and *M*_w_) and dispersity of the melt-pressed starting materials were determined using a gel-permeation-chromatography (GPC) system equipped with a Waters^TM^ Alliance 2690 high-performance liquid chromatography (HPLC) separations module, a Waters^TM^ 484 tunable absorbance detector operating at 265 nm, an online multiangle laser light scattering (MALLS) detector fitted with a 20-mW gallium-arsenide laser operating at 690 nm (miniDAWN^®^, Wyatt Technology, Santa barbara, CA, USA), an interferometric refractometer (Optilab® DSP, Wyatt Technology, Santa barbara, CA, USA) operating at 35 °C and 690 nm, and two PLgels (Polymer Laboratories, Amherst, MA, USA) MIXED E GPC columns (pore sizes: 50–103 Å; bead size: 3 µm) connected in series. Tetrahydrofuran (THF) flowing at 1 mL min^−1^ was used as the mobile phase. The specimen concentrations were approximately 5–10 mg mL^−1^ in 100 µL of freshly injected distilled THF. The detector signals were recorded simultaneously, and the absolute molecular weights and Đ were computed using ASTRA^®^ 4.0 software (Wyatt Technology, Santa barbara, CA, USA).

### 2.7. Hydrolytic Degradation Measurements

All the blend specimens with length, width, and thickness of 3, 3, and 0.05 cm, respectively, were hydrolytically degraded at 37 ± 0.2 °C in a 0.1 N sodium hydroxide (NaOH, pH 13) solution following the accelerated hydrolytic degradation method by Wang et al. [37]. All the fractured blend specimens were carefully weighed before degradation and then dipped into 200 mL NaOH solution to hydrolytically degrade with stirring for 6 days. The degraded specimens were then removed, washed with fresh water, dried in chamber containing a desiccant to completely remove any residual moisture, and reweighed. The same procedure was repeated several times. The final degradation, *F* (wt.%), is given by Equation (1) as follows:

*F* = [(*W_0_ − W*_1_)*/W*_0_] × 100, (1)

where *W*_0_ represents the initial weight (g) of the blend specimen before hydrolysis, and *W*_1_ is the residual weight (g) of the specimen degraded. The hydrolytic degradation was measured three times for each specimen, and the data closest to the average degradation were reported.

### 2.8. Thermal-Property Analysis

Differential scanning calorimetry (DSC) measurements were performed using a DSC-Q20 calorimeter (TA Instruments, Milford, MA, USA). The specimens were heated at 10 °C min^−1^ and scanned several times under nitrogen in the range from −30 to 230 °C. The melting temperatures (*T*_m_) were determined from the main peaks in the initial DSC curves. The glass-transition temperatures (*T*_g_) were calculated based on the midpoints of the change in the heat capacity, as indicated in the DSC scans.

The copolyester specimens were maintained at 90 °C to remove any residual moisture and then heated at 10 °C min^−1^ to 800 °C under nitrogen, and the thermal stabilities of the specimens were studied using a TGA 4000 thermogravimetric analyzer (PerkinElmer, Waltham, MA, USA).

The specimens were melted at 190 °C for 300 s with a melt-index flowmeter (WL1400, WithLab, Seoul, Korea) and then pushed with a 2.16-kg bar for 60 s.

## 3. Results and Discussion

### 3.1. FTIR Analysis

**Figure 2:**
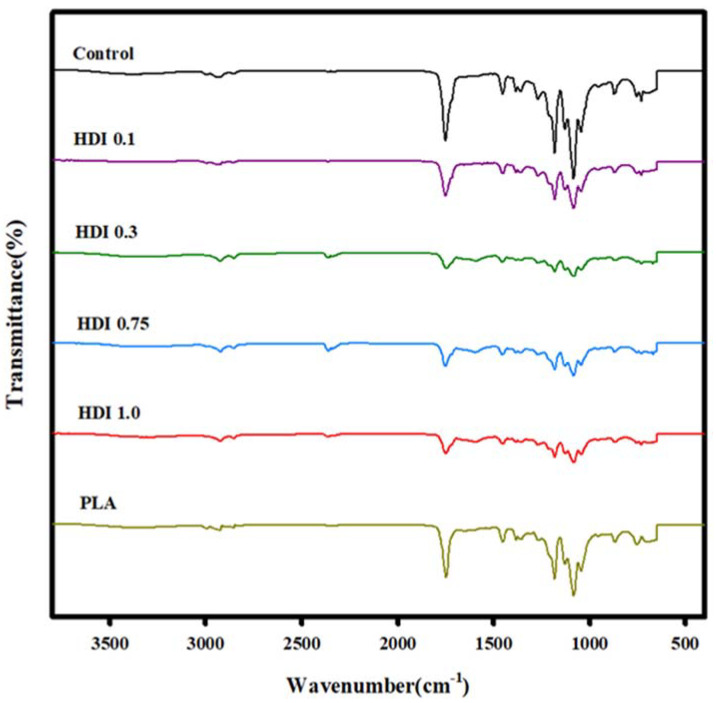
FTIR spectra of neat PLA, control, and blend specimens prepared with various HDI contents.

The FTIR spectra of the neat PLA, control, and blends prepared with various HDI contents are shown in Figure 2. A peak corresponding to the –CH_3_ of PLA at 2945 cm^−1^, 2996 cm^−1^ [38] and the –CH– of PLA at 2997 cm^−1^ (asym), 2946 cm^−1^ (sym), and 2881 cm^−1^ stretch [39]. The –CH_2_ of PBSeT was appeared at 2919 cm^−1^, 2851 cm^−1^ [36]. However, it occurred nearby each other, so whole peaks were appeared in the range 2800–3000 cm^−1^ as small round curves. In addition, the peaks at approximately 1700 and 1180 cm^−1^ correspond to the carbonyl C=O and C–O vibrations, respectively [40,41,42]. In the spectra of both PBSeT and PLA, peaks in the 1700 and 2800–2900 cm^−1^ regions correspond to the C=O bond and aliphatic –CH_2_ stretching, respectively [43]. The spectrum of PBSeT mainly shows peaks in the 1200 and 1100 cm^−1^ regions corresponding to the asymmetric and symmetric stretching of aromatic CO, respectively. In particular, the spectra of all the PBSeT/PLA blends showed a peak in the 720 cm^−1^ region corresponding to the aromatic group, indicating that the PBSeT and PLA had blended well [44]. With increasing HDI content, peaks corresponding to –C=OO^−^ were observed in the 1590 and 660 cm^−1^ regions. In particular, the peak at 1590 cm^−1^ (attributed to NH bending) indicates that the –N=C=O group had changed into the –NH–C=OO^−^ one [32,45]. In addition, a peak attributed to NH stretching appeared in the region at approximately 3300 cm^−1^ when –N=C=O groups were bonded [32,46], and the intensity of the peak at approximately 2300 cm^−1^ (corresponding to the HDI isocyanate group) was very weak (i.e., 0.1). Furthermore, the spectrum for the HDI 0.3 blend specimen clearly showed peaks attributed to the HDI isocyanate group [47], suggesting that the HDI had reacted appropriately with the PBSeT and PLA.

### 3.2. GPC Analysis

**Figure 3:**
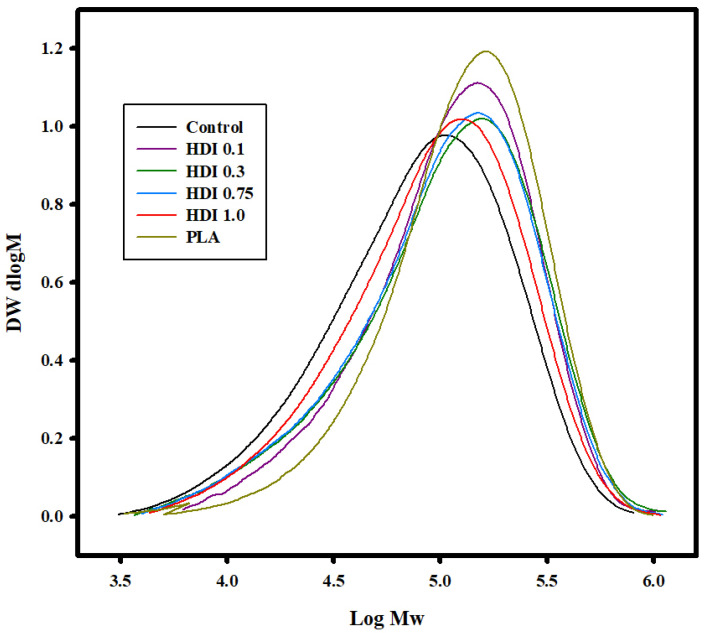
GPC curves of control and blend specimens prepared with various HDI contents.

Table 2 and Figure 3 shows the GPC curves used to determine the *M*_w_, *M*_n_, and Đ of the control and blends prepared with various HDI contents. The control and all the blend specimens showed *M*_w_ values in the range ~160,000 to 170,000 g mol^−1^, lower than the known *M*_w_ of PLA. In addition, PBSeT showed *M*_w_ and *M*_n_ of 154,900 and 64,600 g mol^−1^, respectively. The control showed both the lowest *M*_w_ and *M*_n_ molecular weights of 114,000 and 45,400 g mol^−1^, respectively, which were remarkably lower than those of all the HDI specimens, suggesting that the HDI had increased the molecular weights of the specimens. The control and the HDI 0.1, 0.3, 0.75, and 1.0 specimens showed *M*_w_ molecular weights of 114,000; 148,200; 152,000; 146,300; and 131,300 and *M*_n_ molecular weights of 45,400; 67,800; 57,100; 56,800; and 54,400 g mol^−1^, respectively. The HDI 0.3 specimen showed the highest molecular weight and, therefore, high tensile strength and elongation in the tensile-property analysis. The results suggest that the physical properties of the blends were improved by adding HDI. Moreover, the additional peaks in the FTIR spectra of the HDI specimens suggest that the molecular weights increased owing to chemical bonding.

### 3.3. Tensile Strength and Elongation Properties

**Figure 4:**
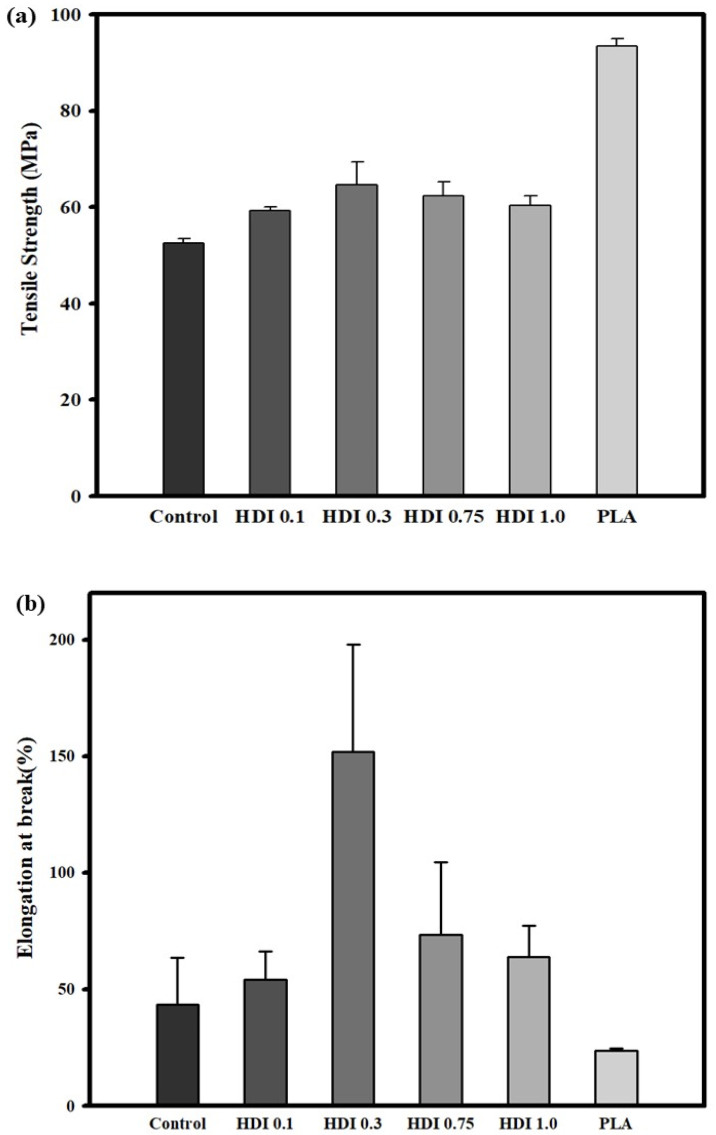

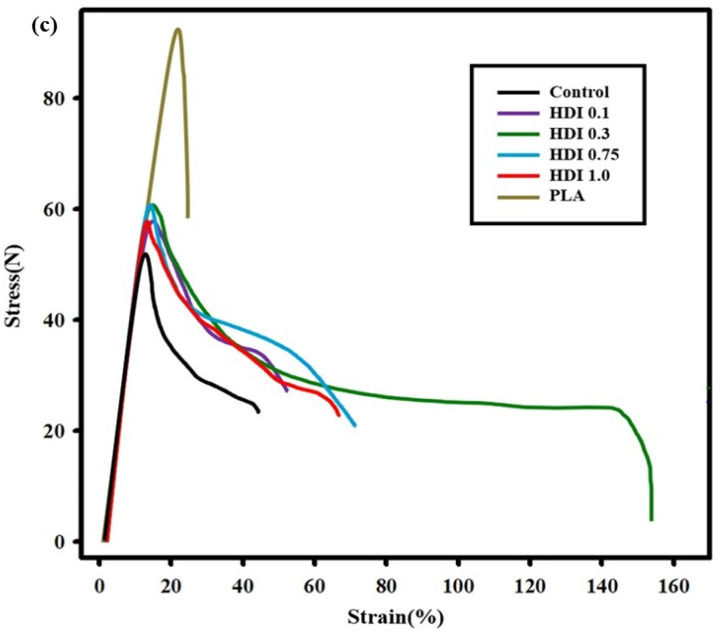
Tensile strength (**a**), elongation (**b**) characteristics and stress–strain curves (**c**) of neat PLA and blend specimens prepared with various HDI contents.

Figure 4 and Table 3 shows the tensile-strength characteristics and stress–strain curves of the neat PLA, control, and blend specimens prepared with various HDI contents. According to previous research results, because PBSeT showed high elongation, it was expected to remarkably affect the tensile properties of the highly brittle PLA [36]. The neat PLA and simple-blend control specimens showed tensile strengths of approximately 93 and approximately 52 MPa, respectively. The results of the one-way analysis of variance (ANOVA) with *post*-*hoc* Tukey honestly significant difference (HSD) showed a statistically significant difference between the tensile strengths of the control and PLA specimens. Furthermore, the control and PLA specimens showed elongations of approximately 43 and approximately 23%, respectively. However, because the standard deviation of the control specimen was relatively high in the significance validation, the difference between the elongations was negligible. The tensile strengths of all the HDI specimens were significantly different from that of the control specimen. However, among the HDI specimens, only the HDI 0.3 one showed a significantly different tensile strength of 64 MPa.

The average tensile strengths of the neat PLA and HDI 0.1, 0.3, 0.75, and 1.0 specimens were 52, 59, 64, 62, and 60 MPa, respectively. Although the average tensile strength of the HDI 0.3 specimen was not significantly different from that of the HDI 0.75 one, it was significantly different from those of all the other specimens. In addition, the HDI 0.3 specimen remarkably showed approximately 151% elongation, which was significantly different from the elongations of all the other specimens. The neat PLA specimen showed the lowest elongation as 23%, and the elongations of the control, HDI 0.1, 0.3, 0.75, and 1.0 specimens were 43, 54, 151, 73, and 63% of elongation, respectively. Therefore, although adding HDI increased specimen elongation up to a point similar to the findings of Kim et al. (2012) [48], adding more than 0.75 phr of HDI was an overdose, which decreased the specimen elongation.

Interestingly, with increasing HDI content from 0.3 to 1.0 phr, the specimen elongation and tensile strength both decreased, suggesting that 0.3 phr was the most appropriate HDI content for increasing the tensile strength and elongation of the blend specimens. Because HDI contents of 0.75 phr or higher caused binding reactions such as self- and branch bonding, the HDI 0.75 and 1.0 specimens did not exhibit appropriately high tensile strength or elongation, which may be why the HDI 0.75 and 1.0 specimens showed two melting temperature (*T*_m_) peaks in the DSC thermal analysis, as will be further discussed in Section 3.5. Furthermore, such binding reactions may be why the HDI was not well dispersed throughout the matrixes of the HDI 0.75 and 1.0 specimens and, thus, why sufficient binding was not achieved. This finding means that when an appropriate amount of modifier is added to the blend, many functional sites can react and be efficiently combined with the modifier. However, it is expected that when a large amount of HDI is added, the binding efficiency is lowered because the HDI cannot be combined with all the functional sites, which had already reacted with each other.

Although the specimen strength and elongation both eventually increased owing to HDI-induced bonding, the two materials were not completely bonded together. Although HDI contents above 0.3 phr were excessive, the HDI is highly reactive and appeared to react quickly wherein all the reaction sites reacted with each other. Therefore, additional investigations (wherein the screw combination and velocity and temperature are varied) are required to elucidate the bonding mechanism and optimize the blending method.

Figure 4 was the stress–strain curves show that except for the neat PLA specimen, all the HDI blend specimens showed improved strains owing to the high PBSeT stretchability. However, some specimens showed necking, which is thought to occur past the PLA yield point, owing to the PBSeT, and the average strains of the HDI blends were slightly different depending on the HDI content.

Necking past the yield point and the phenomena leading to fracture are indicated by the initial slope in the stress curve as steep as the slope the PLA tensile-strength curve before the appearance of the PBSeT stretchability and ductility characteristics. The control, which consisted of only PBSeT blended with PLA, showed improved average strain compared with that of the neat PLA. Furthermore, adding HDI increased the average strains compared with those of the neat PLA and control specimens. In particular, the HDI 0.3 specimen showed the highest strain of approximately 150%.

### 3.4. Three-Point Flexural Strengths

**Figure 5:**
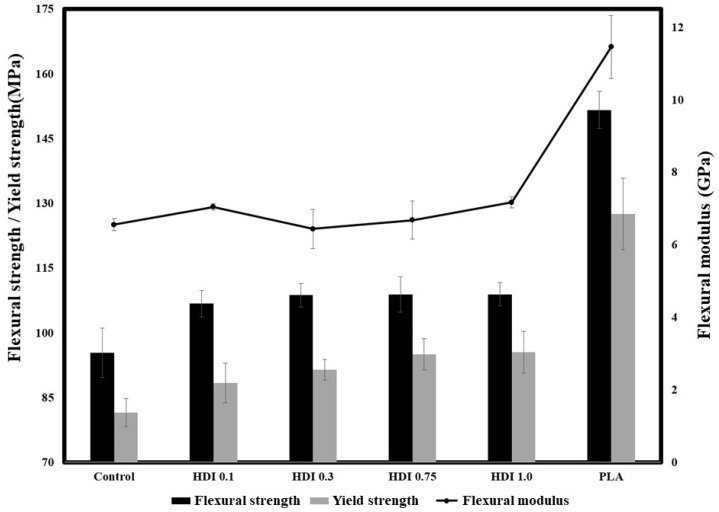
Three-point flexural strengths and moduli of neat PLA, control, and blend specimens prepared with various HDI contents.

Figure 5 and Table 4 shows the 3-point flexural strengths and moduli of the neat PLA, control, and blend specimens prepared with various HDI contents. When PBSeT was blended with neat PLA, the flexural strength decreased from approximately 150 (for the neat PLA) to 95 MPa (for the blended control), suggesting that the relatively brittle PLA was made more ductile by adding the elastomeric PBSeT. However, all the HDI specimens showed higher flexural strengths than the control. Specifically, the HDI 0.1, 0.3, 0.75, and 1.0 specimens exhibited flexural strengths of 106, 108, 108, and 108 MPa, respectively, which although were higher than that of the control, were not remarkably different. However, the control and HDI 0.1, 0.3, 0.75, and 1.0 specimens showed yield strengths of approximately 81, 88, 91, 95, and 95 MPa, respectively. Although the yield strength increased with increasing HDI content, the yield strengths of the HDI 0.75 and 1.0 specimens were not remarkably different. The flexural strengths of all the HDI specimens are similar despite the increased yield-point strength because the experiment was conducted according to the ISO standard method of terminating the experiment when the strain reached 5%. Although the maximum flexural strengths of the specimens appeared similar up to a certain point, the yield-point strength slightly increased with increasing HDI content. In particular, the flexural modulus of the HDI 0.1 specimen was approximately 7 GPa, which is higher than that (6.5 GPa) of the control specimen, indicating that the HDI had increased the momentary modulus. However, the HDI 0.3, 0.75, and 1.0 specimens showed flexural moduli of 6.4, 6.6, and 7.1 GPa, respectively, indicating that the flexural modulus slightly decreased and then increased with increasing HDI content and that the HDI 0.3 specimen showed the maximum elongation of approximately 150%. Although the HDI 0.3 specimen showed a somewhat decreased flexural modulus, the yield-point strength was not reached during a longer strain, suggesting that the HDI 0.3 specimen could withstand strain without fracturing.

### 3.5. Thermal Properties

**Figure 6:**
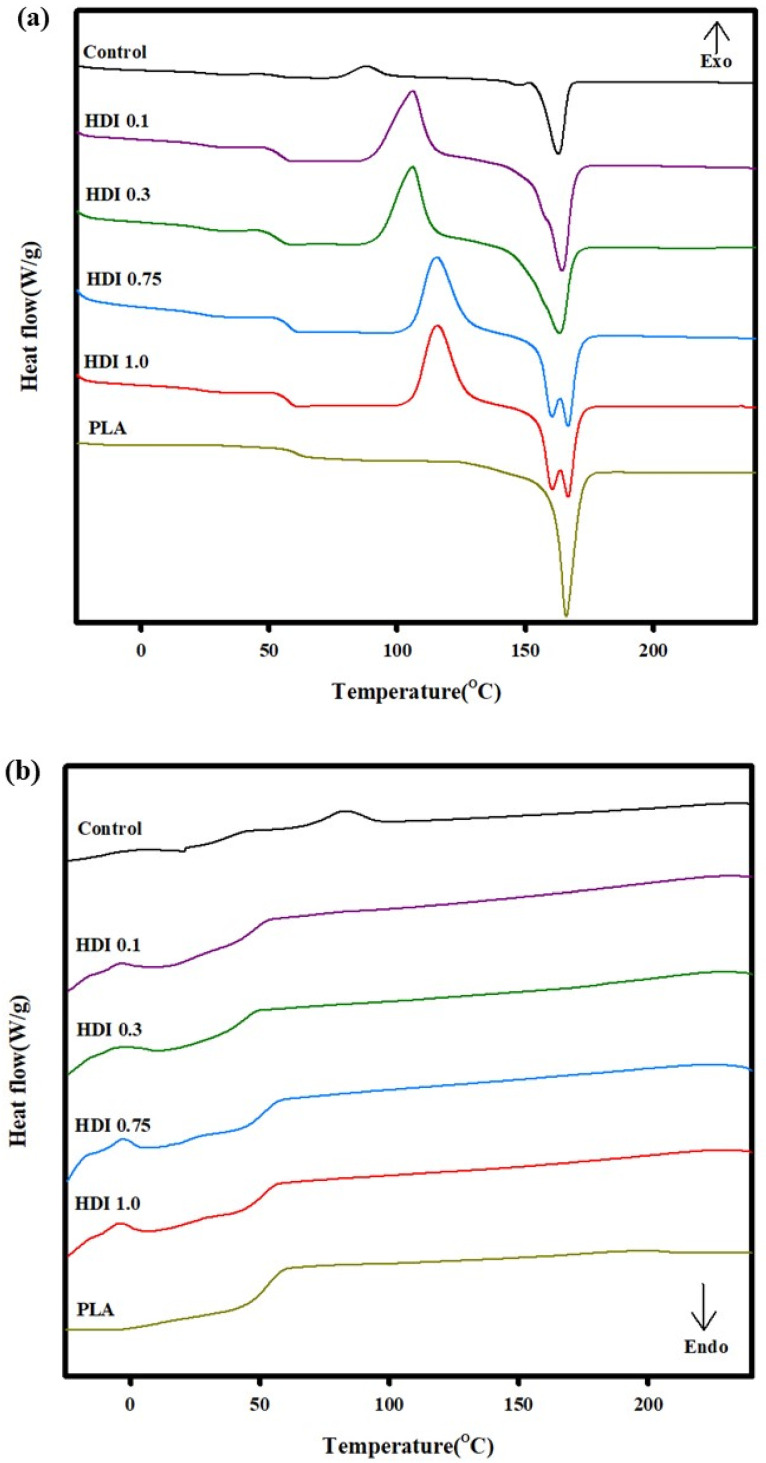
DSC thermal characteristics of control and blend specimens prepared with various HDI contents, (**a**) 2nd heating and (**b**) 2nd cooling.

The thermal properties of the control and blend specimens were investigated by DSC. The DSC curves of the specimens heated a second time (i.e., the “second heating curves”) were used to determine the melting points and characterize the thermal properties of the specimens because the second heating curves provide more-accurate data independent of the thermal histories of the specimens. Figure 6 shows the DSC curves of the control and blend specimens prepared with various HDI contents, and Table 5 shows *T*_g_, the cold-crystallization temperature (*T*_cc_), *T*_m_, and the other thermal properties of the specimens. A peak corresponding to *T*_g_ appeared in the DSC curves of all the specimens, and the peak shifted depending on the HDI content of the specimen. The DSC curve of the control showed a *T*_g_ peak at approximately 53 °C, which is slightly lower than the *T*_g_ previously known for neat PLA. The DSC curve of the control also showed a peak corresponding to *T*_cc_ at 87 °C, which is lower than the *T*_cc_ of the other specimens, indicating a fairly low *T*_cc_. It previously was thought that PBSeT affected the PLA crystallization rate and that the PLA cold-crystallization heat capacity was 6.2 J g^−1^, which is lower than those of the other specimens. However, in the current work, although the *T*_cc_ of the control was 87 °C, the *T*_cc_ peak shifted to higher temperatures and the peak sizes increased with increasing HDI content. For example, although the *T*_cc_ of the HDI 0.1 and 0.3 specimens was approximately 105 °C, that of the HDI 0.75 and 1.0 ones increased to approximately 115 °C, which is associated with increased crystallinity and is thought to originate from the increasing size and number of crystals with increasing HDI content. This finding suggests that the HDI and PBSeT contents both affect the crystallization of the main PLA domain because HDI and PBSeT both are almost amorphous and in particular, it suggests that the HDI-centered crystal structure affects the PLA crystallization.

The control and HDI 0.1, 0.3, 0.75, and 1.0 specimens, showed 7, 18, 30, 31, and 32% crystallinity, respectively. Although the crystallinity increased with increasing HDI content and the crystallinity of the HDI 0.1 specimen did not increase significantly to 18%, the crystallinity of the HDI 0.3 specimen did significantly increase to 30%. However, the crystallinities of HDI 0.75 and 1.0 specimens were not significantly different.

The control specimen showed the lowest *T*_m_ of 163 °C, and a relaxation zone appeared before *T*_m_ in the DSC second-heating curve of the control owing to insufficient PBSeT and PLA mixing. However, the relaxation zone disappeared when HDI was added to the specimens. The *T*_m_ of all the specimens were measured in the range ~163–167 °C and were all slightly lower than the *T*_m_ previously known for neat PLA, except HDI 0.75 and 1.0 which showed two *T_m_* peaks.

The DSC second-heating curves of the HDI 0.75 and 1.0 specimens showed similar *T*_m_ peaks at 159 and 167 °C, respectively, unlike the curves of the other specimens. The *T*_m_ measured for the neat PLA is affected by either changes in the PLA crystal shapes and sizes owing to the addition of a nucleating agent or by the increasing thicknesses of external and internal crystals. In this study, the *T*_m_ was affected by the formation of additional crystals when HDI was added to the specimens.

The mechanical and physical properties of blends consisting of an amorphous polymer (such as PBSeT) blended with a semicrystalline polymer (such as PLA) are greatly affected by the crystallization kinetics, degree of crystallinity, and microstructure of each of the individual blends in the same structure. The miscibility, properties, and internal structural interactions of amorphous/semicrystalline blends greatly influence the crystallinity and physical strength. Usually, the higher the polymer crystallinity is, the higher the physical stiffness (i.e., elasticity modulus) and tensile strength are, which decrease ductility. Although most of the specimens in this study followed that general rule, the HDI 0.3 specimen showed drastically increased tensile strength and elongation.

**Figure 7:**
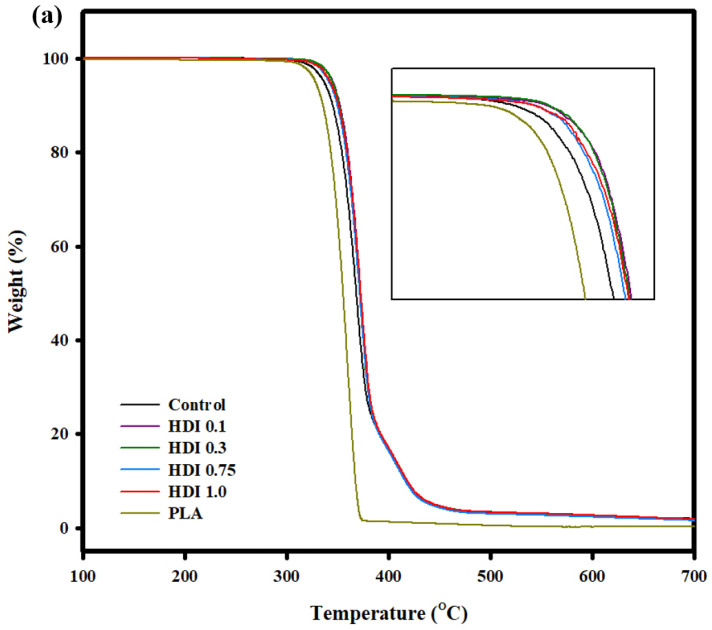
(**a**) TGA and curves of PLA and PLA/PBSeT blends prepared with various HDI contents.

Figure 7 shows the TGA curves of the control and blend specimens prepared with various HDI contents. All the specimens were sufficiently dried before testing, and none of them showed any weight loss at approximately 100 °C. All the specimens were pyrolyzed above approximately 340 °C, and the TGA curves of all the specimens exhibited peaks indicating two-stage decomposition because PLA and PBSeT show different decomposition temperatures. Previously reported main decomposition temperature of PBSeT was around 400 °C [36].

However, the main peaks of all the HDI specimens were at decomposition temperatures higher than that of the main peak of the control (PBSeT/PLA blend) specimen. That is, the control specimen mainly decomposed at approximately 370 °C, while the HDI 0.1, 0.3, 0.75, and 1.0 specimens decomposed at 372, 371, 372, and 371 °C, respectively. The decomposition and decomposition-onset temperatures of the HDI specimens were not remarkably different from those of the control specimen, indicating that the HDI had only slightly changed the heat resistance of the blends.

### 3.6. Hydrolytic Degradation

**Figure 8:**
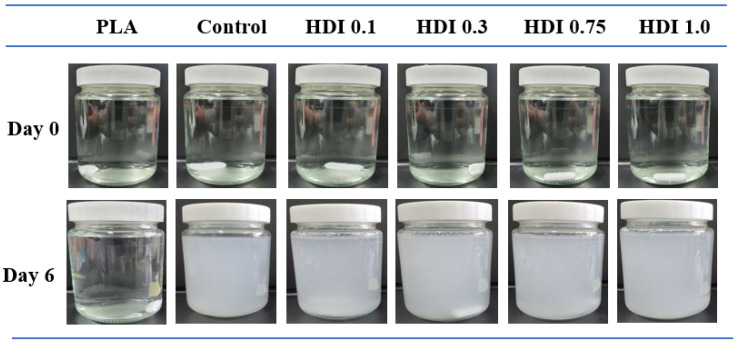
Appearances of neat PLA, control, and blend films prepared with various HDI contents and immersed in NaOH solution at 0 and 6 days.

PLA based blends previously have been hydrolyzed under accelerated conditions to demonstrate its biodegradability. For example, Wang et al. studied PLA biodegradability by hydrolyzing PLA–PBS blends in NaOH [37]. Furthermore, Heidarzadeh et al. reported that PBSeT was biodegradable [43]. Therefore, because each of the individual polymer components is biodegradable, the PLA/PBSeT blends are also expected to be biodegradable. Moreover, the GPC data revealed that the molecular weights of all the HDI specimens were higher than that of the HDI-free control. Table 6 represents the weight-loss-based degradation ratios determined for the neat PLA and blend specimens prepared with various HDI contents. The appearances of the corresponding films immersed in NaOH solution at 0 and 6 days are shown in Figure 8. All film specimens were hydrolytically degraded in NaOH. Interestingly, all the specimens except for the neat PLA showed at least 65% degradation when immersed in NaOH solution at 37 °C for 6 days. The HDI-free control specimen decomposed surprisingly quickly, showing over 90% decomposition at 144 h. Because the HDI increased the molecular weights and cross-linking of PLA/PBSeT blends, all the HDI added specimens degraded slightly less than the control. These fast hydrolytic degradation of PLA/PBSeT blends could be accelerated also by mechanical stirring process. However, the neat PLA steadily degraded, showing approximately 10% degradation after 144 h. The neat PLA, which is digestible in thermophile condition, decomposed slower than expected. The PLA is main film matrix and PBSeT acts as the dispersed component, where 20% PBSeT are degraded first in mesophilic temperature (37 °C) causing the disintegration of film specimens. Despite the blended blends containing 80% PLA and only 20% PBSeT, they all showed highly accelerated decomposition. However, it is particularly noteworthy that increasing the molecular weight of the blends did not proportionally increase their degradability, indicating that the difference in molecular weights was negligible in determining the rate and degree of blend decomposition.

## 4. Conclusions

In this study, synthesized PBSeT and neat PLA were blended with various contents of HDI (a crosslinking agent) to increase the blend miscibility, and the mechanical and chemical properties of the blends were investigated. All specimens degraded significantly faster than the neat PLA specimen and showed high degradabilities of over 65%. In particular, the HDI-free control specimen showed over 90% degradation. Because elastomeric PBSeT showed very high elongation in previous studies, we utilized its viscoelastic characteristics by combining PBSeT with PLA, and the resulting PBSeT–PLA blend showed elongation higher than that of neat PLA. All the HDI blends showed tensile strengths and elongations higher than those of the control. In particular, the HDI 0.3 specimen showed remarkably high elongation and the highest tensile strength of all the specimens, suggesting that 0.3 phr was the appropriate HDI content for optimizing the physical properties of PLA. The results of this study suggest the use of the obtained blends in flexible packaging industry.

## Figures and Tables

**Table 1:** Chemical compositions of PLA/PBSeT blends prepared with various HDI ratios.

Specimen	PLA% (*w/w*)	PBSeT% (*w/w*)	HDI (phr)
Control	80	20	0
HDI 0.1	80	20	0.1
HDI 0.3	80	20	0.3
HDI 0.75	80	20	0.75
HDI 1.0	80	20	1.0

**Table 2:** Polymer molecular weight and dispersity (M_w_/M_n_) of PLA/PBSeT blends.

Unit		Control	HDI 0.1	HDI 0.3	HDI 0.75	HDI 1.0
M_w_	g/mol	114,000	148,200	152,000	146,300	131,300
M_n_	g/mol	45,400	67,800	57,100	56,800	54,400
*Đ* (Dispersity)	M_w_/M_n_	2.5	2.1	2.6	2.5	2.4

**Table 3:** Tensile strength and elongation at break PLA/PBSeT based blends with various HDI contents.

	Control	HDI 0.1	HDI 0.3	HDI 0.75	HDI 1.0	PLA
Tensile strength (MPa)	52.6	59.34	64.64	62.36	60.4	93.36
STDV	0.8	0.6	4.8	2.9	1.8	1.6
Elongation at break (%)	43.34	54.15	151.9	73.36	63.78	23.58
STDV	20.2	12.0	46.1	31.0	13.4	0.9

**Table 4:** Flexural properties (strength, modulus, and yield strength) of PLA/PBSeT based blends with various HDI contents.

	Control	HDI 0.1	HDI 0.3	HDI 0.75	HDI 1.0	PLA
Flexural Strength (MPa)	95.3	106.7	108.7	108.9	108.9	151.6
Flexural Modulus (GPa)	6.5	7.0	6.4	6.6	7.1	11.4
Yield Strength (MPa)	81.5	88.3	91.4	95.0	95.4	127.5
F.S. STDV	5.7	3.0	2.6	4.1	2.7	4.2
F.M. STDV	0.1	0.1	0.5	0.5	0.1	0.8
Y.S. STDV	3.2	4.6	2.3	3.6	4.8	8.2

**Table 5:** DSC data of the PLA/PBSeT blends with various HDI contents.

	Control	HDI 0.1	HDI 0.3	HDI 0.75	HDI 1.0	PLA
*T_m_* (°C)	163.3	164.5	163.3	159.9/167.1	159.9/167.1	166.1
ΔHf (J/g)	21.8	25.2	34.5	31.6	32.9	26.0
*T_g_* (°C)	53.4	54.6	53.9	58.2	58.0	59.1
*T_cc_* (°C)	87.9	105.7	105.9	115.4	115.5	-
ΔHcc (J/g)	6.4	16.8	28.4	29.3	23.4	-

**Table 6:** Weight-loss-based degradation ratios determined for neat PLA, control, and blend specimens prepared with various HDI contents all hydrolytically degraded in NaOH.

		Time of Degradation										
		24 h	36 h	48 h	60 h	72 h	84 h	96 h	108 h	120 h	132 h	144 h
Weight loss (%)	Control	7.3	18.0	37.4	44.0	49.4	55.6	68.0	76.0	79.8	83.8	92.1
	HDI 0.1	8.0	14.1	18.4	24.2	35.7	42.6	50.3	55.6	61.4	64.7	67.5
	HDI 0.3	10.1	24.9	27.6	31.9	38.0	53.4	58.0	64.1	69.8	77.6	85.9
	HDI 0.75	10.6	18.8	25.6	29.6	40.2	51.0	57.2	62.2	67.6	73.4	78.6
	HDI 1.0	9.9	17.8	28.2	31.9	38.1	46.4	51.1	59.8	65.8	70.5	71.8
	PLA	1.0	1.9	2.8	3.3	4.6	4.7	5.0	7.2	8.4	9.2	10.3

## Data Availability

Data sharing is not applicable to this article.
